# Gastrointestinal epithelial innate immunity—regionalization and organoids as new model

**DOI:** 10.1007/s00109-021-02043-9

**Published:** 2021-02-04

**Authors:** Özge Kayisoglu, Nicolas Schlegel, Sina Bartfeld

**Affiliations:** 1 Research Centre for Infectious Diseases, Institute for Molecular Infection Biology, Julius Maximilians University of Wuerzburg, Wuerzburg, Germany; 2 Department of General, Visceral, Transplant, Vascular and Pediatric Surgery, University Hospital Wuerzburg, Oberduerrbacher Strasse 6, Wuerzburg, Germany

**Keywords:** Gastrointestinal tract, Immunity, Regionalization and organoids

## Abstract

The human gastrointestinal tract is in constant contact with microbial stimuli. Its barriers have to ensure co-existence with the commensal bacteria, while enabling surveillance of intruding pathogens. At the centre of the interaction lies the epithelial layer, which marks the boundaries of the body. It is equipped with a multitude of different innate immune sensors, such as Toll-like receptors, to mount inflammatory responses to microbes. Dysfunction of this intricate system results in inflammation-associated pathologies, such as inflammatory bowel disease. However, the complexity of the cellular interactions, their molecular basis and their development remains poorly understood. In recent years, stem cell–derived organoids have gained increasing attention as promising models for both development and a broad range of pathologies, including infectious diseases. In addition, organoids enable the study of epithelial innate immunity in vitro. In this review, we focus on the gastrointestinal epithelial barrier and its regional organization to discuss innate immune sensing and development.

## Introduction

**Fig. 1:**
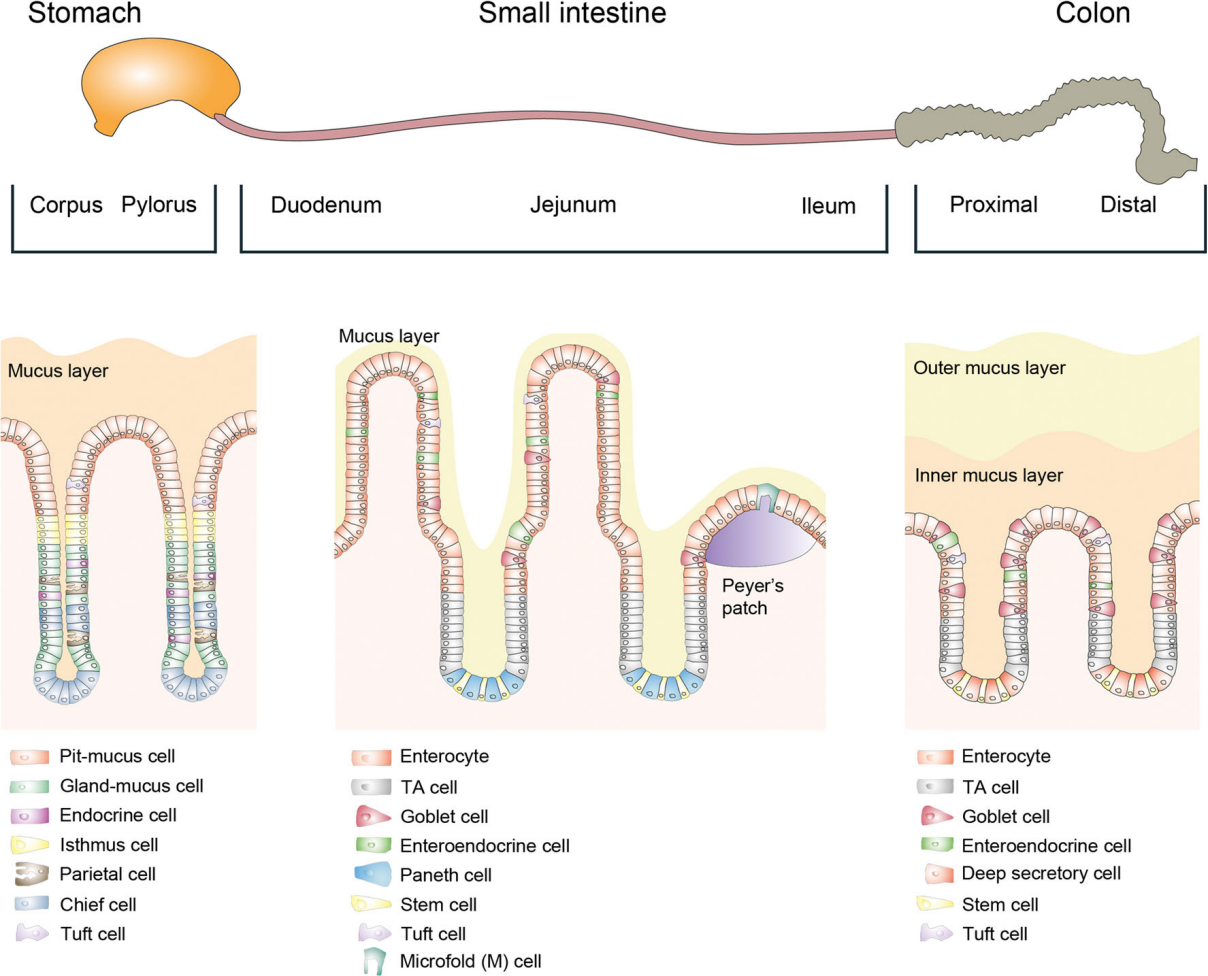
Overview of the gastrointestinal epithelium. The gastrointestinal epithelial layer is organized into invaginations called glands in the stomach, and crypts of Lieberkühn in the intestine. In the small intestine, villi protrude into the lumen to maximize contact for nutrient uptake. Likely, also to counteract the frequent infections or the surface cells, the body heavily invests in the turnover of cells, specifically at the surface of the invaginations. In the stomach, as well as in the small and large intestine, adult stem cells reside within the invaginations and constantly proliferate. They produce further proliferating undifferentiated progenitor cells (transit-amplifying cells in the intestine, isthmus cells in the stomach). The cells below this region of amplification have a relatively long lifetime: Small intestinal Paneth cells, residing at the base of the crypt, have a lifespan of about 3–6 weeks. Similarly, gastric glandular cells, such as chief cells, residing below the isthmus region have a lifespan of several months. In contrast, cells above the amplification region move conveyor belt-like towards the surface, finally reaching the gastric pit, the small intestinal villus or the colon crypt opening, where they are shed into the lumen after a lifetime of only 3–5 days [4–6]. Both organs have absorptive enterocytes and secretory cells, such as mucus-producing goblet cells, hormone-producing enteroendocrine cells and tuft cells. In addition, the small intestinal epithelium also contains Paneth cells, which produce antibacterial peptides, as well as specialized microfold (M) cells on the Peyer’s patches, enabling crosstalk between the microbiota and the immune system.

The gastrointestinal (GI) tract is required for the digestion of food and spans from the oral cavity via the oesophagus, stomach, small intestine and large intestine to the anus. The GI lumen is colonized by a vast variety of commensals, symbionts and occasionally pathogens. The microbial colonization follows a gradient with less than 10^3^ microbes/ml in the stomach to 10^3^–10^7^ microbes/ml in the small intestine and 10^11^–10^12^ microbes/ml in the colon (reviewed in [1–3]). From the glandular stomach onwards, the GI tract is lined by a single layer of columnar epithelial cells. This epithelial layer comprises of different specialized cells, tightly interlocked by junctional protein complexes, enforcing the physical barrier (Fig. 1).

**Fig. 2:**
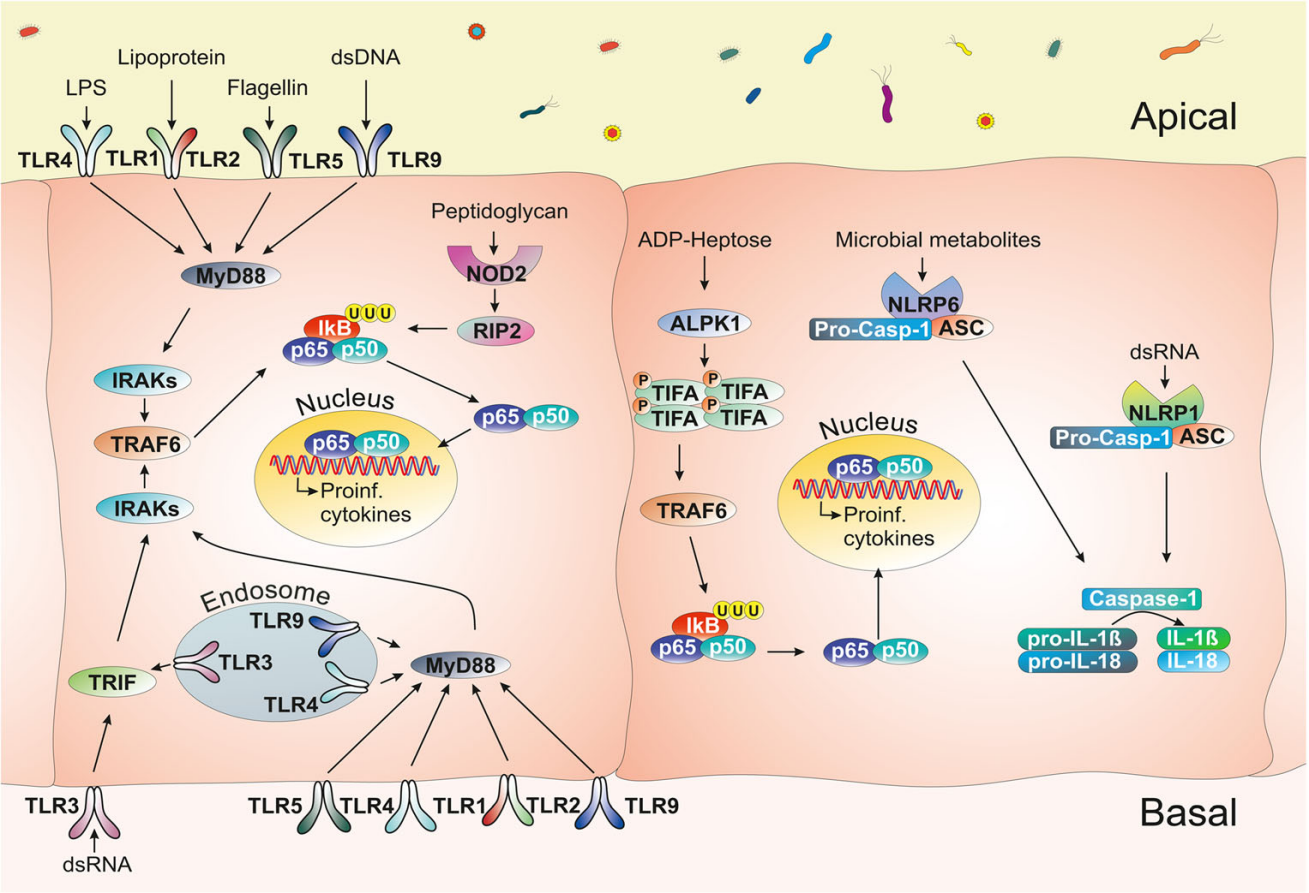
Simplified overview of PRR signalling pathways in gastrointestinal epithelial cells. Pattern recognition receptors (PRRs) including TLRs, NLRs or newly discovered sensors such as ALPK1 recognize the microbe-associated molecular patterns (MAMPs). Upon PRR activation in epithelial cells, downstream signalling cascades induce expression of different cytokines and chemokines via inflammatory pathways, such as the NF-κB pathway. TLR1, 2, 3, 4, 5 and 9 recognize lipoproteins, double-stranded (ds-) RNA, lipopolysaccharides (LPS), flagellin and dsDNA, respectively. ALPK1 recognizes the LPS metabolite ADP-heptose and its stimulation leads to phosphorylation of TIFA proteins, mediating the formation of TIFAsomes as a response to gram-negative bacteria. Ligands bind to a receptor which leads to the recruitment of adaptor proteins (e.g. Myd88, TRAF6 and RIP2). These adaptors drive the phosphorylation of the IκB which leads to its ubiquitination and degradation. NF-κB subunits p65 and p50 can then enter the nucleus to facilitate the expression of target genes which are proinflammatory cytokines such as *IL-8* in humans and its analogue *Cxcl2* in mice. Inflammasomes, which are cytoplasmic complexes composed of NLR proteins, recognize additional molecular patterns, microbial metabolites or nucleic acids. They activate caspase-1, which cleaves and thereby activates proinflammatory cytokines like pro-IL-1b and pro-IL-18, driving the downstream inflammatory pathways. In turn, proinflammatory cytokines will recruit professional immune cells of the innate and adaptive immune system, which are equipped to resolve the infection (reviewed in [7–10]).

The interaction between the gut microbiota as well as ingested pathogens and epithelial cells is mediated by pattern recognition receptors (PRRs), including Toll-like receptors (TLRs), nucleotide-binding oligomerization domain (NOD)–like receptors (NLRs) and other cytosolic receptors (Fig. 2, reviewed in [7, 11]). These PRRs play a key role in recognizing microbe-associated molecular patterns (MAMPs) and damage-associated molecular patterns (DAMPs). Upon PRR activation in epithelial cells, downstream signalling cascades induce expression of different cytokines and chemokines via inflammatory pathways, such as the NF-κB pathway, to direct the professional immune cells (reviewed in [8, 12–16]). In addition to the classical PRRs, further sensors of bacterial activities, such as alpha-kinase 1 (ALPK1) have recently been discovered [17–19]. Inflammasomes, cytoplasmic complexes composed of NLR proteins, recognize additional molecular patterns, such as bacterial metabolites (reviewed in [7, 9]).

Gastrointestinal innate immune responses, including PRR sensing, must balance the need for providing protection from potentially harmful pathogens with being able to tolerate exposure to the diverse luminal microbiome. Hence, the expression and function of PRR signalling are expected to have a major impact not only on pathogen sensing but also on tissue homeostasis (Box 1) and inflammatory diseases including acute gastroenteritis, gastritis and inflammatory bowel diseases (IBDs) (reviewed in [12, 13, 20, 21]). Although each of these diseases displays completely different pathogeneses, they share a critical feature: the interface between the environment and the body is significantly disturbed. However, there is an ongoing debate whether changes of the intestinal epithelial layer, in particular those affecting its innate immune function, are a cause or a consequence of the above-mentioned diseases.

Most knowledge about PRR signalling has been gathered from research on haematopoietic cells. It has been a challenge to discriminate the functions of PRR signalling in epithelial cells from those of the infiltrating immune cells. Major obstacles include difficulties in isolating pure epithelial cells and raising reliable antibodies against PRRs. With the advancement of organoids that derive from intestinal epithelial stem cells, a reductionist experimental model is now available that enables investigations into the innate immune response of the primary epithelial cells. Organoids are defined as stem cell–derived, 3-dimensional cell cultures that have self-organizing capacity and retain some of the function of the original organ (e.g. secretion, filtration, absorption, contraction). Since organoids can be grown either from tissue-resident adult stem cells (ASCs) or from pluripotent stem cells (PSCs), the cells are non-transformed. Together, the two types of organoids cover an extensive repertoire of organs that can be mimicked (reviewed in [4, 22, 23]). Both technologies have their individual advantages (reviewed in [4, 22, 23]). For example, cultures of ASC-derived organoids have a tremendous expansion potential and are relatively homogeneous, and PSC-derived organoids are more complex in the sense that they combine cells of very different developmental origins (e.g. epithelial and mesenchymal cells). PSC-derived organoids allow the analysis of the developmental steps but may not reach the full level of differentiation into epithelial layer as found in vivo [24]. ASC-derived organoids generated from fetal tissues may also allow the study of maturation of fetal epithelia, since they age in culture [24, 25].

This review will highlight insights from studies using organoids and discuss the potential of this technology for the study of epithelial innate immunity. We focus on the regional organization in the gastrointestinal tract.

## Regional identity and innate immune signalling in the GI tract

When looking at gastrointestinal diseases it is important to note that some of them are confined to a specific section of the gastrointestinal tract. IBD includes Crohn’s disease (CD) and ulcerative colitis (UC), which show a differential and disease-specific pattern of inflammation: while UC begins in the rectum and is found in the colon, CD can affect all parts of the GI tract from the oral cavity to the anus. Furthermore, CD is characterized by segmental, discontinuous inflammation within the GI tract, while UC is usually described as a continuous inflammation of the colon (reviewed in [26, 27]). In the oesophagus, stomach and colon, cancer incidence is high, and infection and inflammation can promote development and progression of these cancers (reviewed in [28, 29]). In contrast, malignant transformation in the small intestine is very rare (reviewed in [30]). The apparent segment-specificity of these diseases in the GI tract remains enigmatic, but it is tempting to speculate that their region-specific origin is rooted in region-specific disturbances of the tightly balanced system of epithelial barrier function, innate immunity and mucosal regeneration. Therefore, it is interesting to highlight differences in the segments within the GI tract.

The GI tract comprises several anatomically defined segments with vastly distinct physical functions (reviewed in [31]). The main function of the stomach is the digestion of food and the elimination of incoming pathogens by gastric acid. Nutrients do not have to reach the epithelial layer of the stomach; thus, the body heavily invests in a protective mucus barrier, shielding the epithelial cells not only from its own acid but also from luminal content (reviewed in [32, 33]). By contrast, the main function of the small intestine is not only digestion but also the uptake of nutrients. In keeping with this, the small intestine has a significantly enlarged surface area due to the villi protruding into the gut lumen, where cell surfaces come in close contact with the nutrients. Most of the digestion takes place at the proximal small intestine, duodenum and jejunum, where the villi are long and thin. The jejunum has the highest ratio of Paneth cells secreting antimicrobial peptides [34], which decorate the rather loose mucus, thereby guarding the epithelial layer and keeping the crypts sterile (reviewed in [35]). The villi become progressively shorter and broader towards the ileum (reviewed in [36]), where again, the mucus takes over an important part of the protection: with the highest ratio of goblet cells, the ileum has a thicker mucus layer and a lower rate of digestion and absorption than the jejunum [34] (reviewed in [31]). Lastly, the colon reabsorbs water and invests into an extensive, thick and bi-layered mucus cover to be able to safely harbour trillions of commensal bacteria. The colon has no villi and the crypts are smaller than those of the small intestine. There are no Paneth cells, and the goblet cell ratio can be up to 25% of the epithelial layer [37]. Thus, the three gut segments have different strategies for maintaining a safe distance between the epithelial cells and the microbiota. MAMP recognition and activation of immune pathways is another layer of this interaction and therefore, it is only reasonable that they are also structured along the GI tract (reviewed in [38]). However, it remains a fascinating riddle what exactly shapes the structure of the PRR organization.

While it is intuitive that genes important for regional functions, such as digestion and nutrient uptake, follow spatial compartmentalization along the cephalocaudal axis (reviewed in [39]), such an organization was not expected for epithelial innate immune signalling. Previous studies had reported regulation of PRR signalling in response to stimulation with MAMPs. For example, TLR4 responsiveness decreases after birth, presumably because of the exposure to LPS during delivery and subsequent colonization of the gut [40]. Also, stimulation of TLR9 with its ligand CpG-DNA leads to a decrease of *Tlr4* expression and inhibits TLR4 signalling [41]. Thus, it was expected that contact with the microorganisms, their molecules and metabolites in the GI tract would lead to silencing of PRR expression towards the gut lumen (reviewed in [14]). However, contradicting results, caused by technical difficulties such as unreliable antibodies targeting TLRs, led to confusion in the field and it was unclear, whether a particular PRR was expressed or not (reviewed in [42]). Early Northern blots for mRNA of *Tlr2* and *Tlr4* in ex vivo isolated epithelial cells indicated that expression levels of these two *Tlr* molecules were segment-specific: *Tlr2* was expressed mainly in the colon, while *Tlr4* was mainly expressed in stomach and colon. The authors termed this “strategic compartmentalization” of these TLRs [43]. Recent studies have now discovered that this principle of segment-specific expression extends beyond these two TLRs, and have unravelled a highly complex regional organization of PRR signalling that does not always follow the microbial load [44, 45].

The first of the two studies came from the Barton group and revealed several levels of organization of TLR expression. The group generated five strains of reporter mice, enabling expression analysis of TLR2, 4, 5, 7 and 9, respectively. TLR2 and 5 were expressed in the small intestine and the proximal colon, TLR4 was expressed in colon and TLR7 and 9 were not expressed. Reporter expression in organoids from these mice closely mimicked the in vivo expression, indicating that the expression is independent of contact with the microbiota or with immune cells [45].

**Fig. 3:**
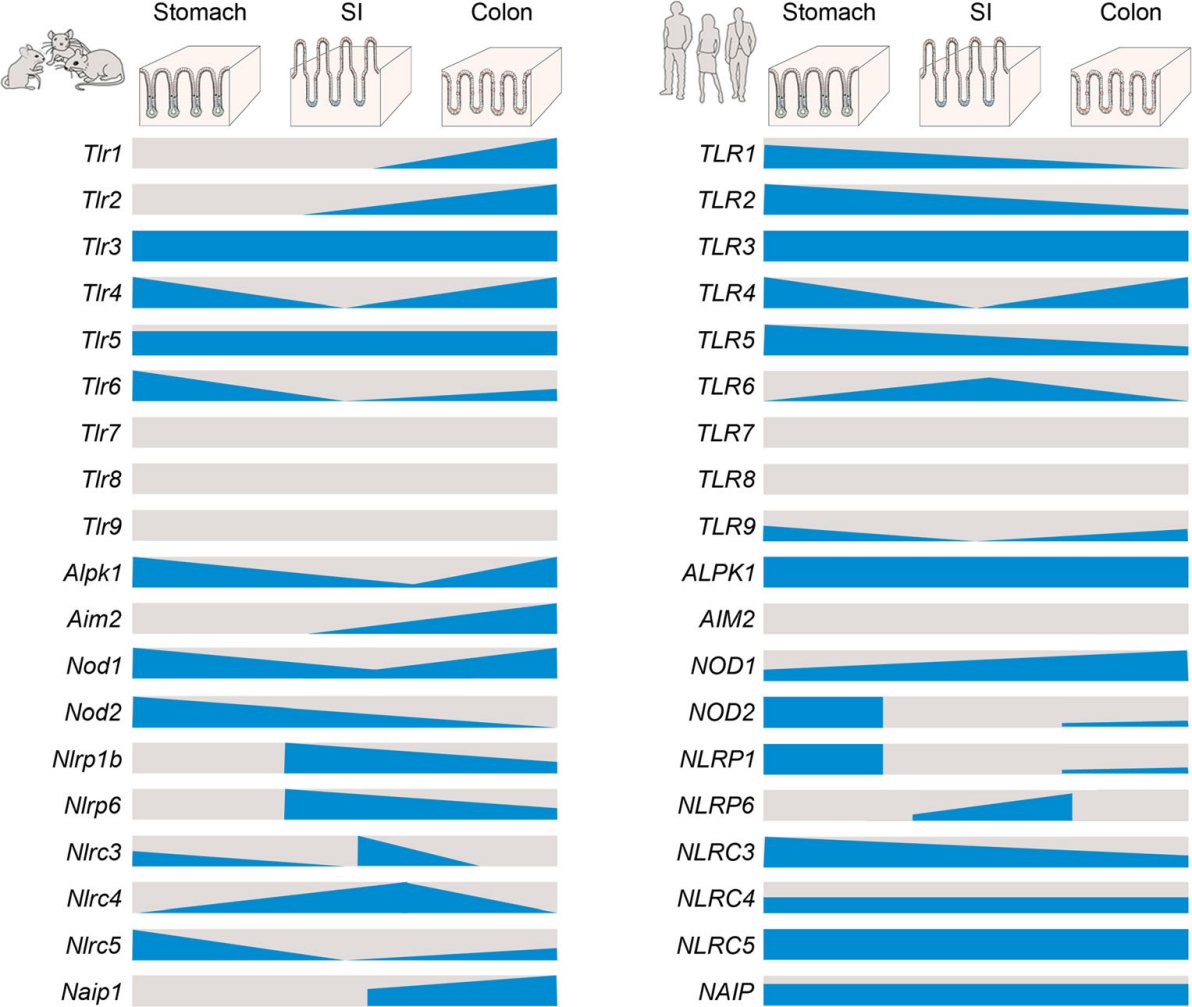
Distribution of various pattern recognition receptors along the murine (left) and human (right) gastrointestinal tract according to a recent study [44]. In this study, organoids were generated from six regions of the GI tract of mice (left) and human (right): the gastric corpus and pylorus, the small intestinal (SI) duodenum, jejunum and ileum and the colon. The graphic illustrates the relative level of RNA expression (blue), as measured by RNA-seq of 3 organoid lines per GI segment. Note the segment-specific expression and the differences between the species.

The second study used a biobank of freshly generated human and murine organoids covering the different segments of the GI tract: corpus, pylorus, duodenum, jejunum, ileum and colon. Transcriptional analysis of the organoids confirmed the expression patterns for the TLRs reported by the Barton group, but in addition revealed a vast extent of differential expression of TLRs, NLRs, inflammasome components and other innate immunity-related genes (Fig. 3 and [44]). For example, *Nod2* was mainly expressed in the stomach, while expressions of several inflammasome pathway components like *Nlrp1b*, *Nlrp6* and *Aim2* were restricted to the intestine. Various receptors were uniformly expressed; for example, *Tlr3* expression was detected in every segment of the murine GI tract in high amounts, whereas *Tlr5* was expressed in every segment but in low amounts [44]. As a result, each segment appears to have its very own, specific complement of innate immune receptors and signalling components.

Comparing the murine with the human gastrointestinal organoids demonstrated that in both species the principle of complex organization of PRR signalling components was the same, but individual PRR expression profiles could differ. Some PRRs, like *TLR4* and *NLRP6*, were similarly patterned along the GI tract of both species. *TLR4* was expressed predominantly in the stomach and colon and *NLRP6* expression was restricted to the segments of the intestine. On the other hand, several PRRs such as *TLR1* and *TLR2* showed patterns that differed between both species. While in murine organoids, expression of both increased along the GI tract and was highest in the colon, in human organoids expression was highest in the stomach and decreased along the GI tract [44]. Moreover, expression of *TLR5* was higher in human organoids, with the highest levels observed in the stomach [44].

As mentioned above, organoids also allow testing of whether a particular pathway is functional by addition of the ligand and subsequent analysis of downstream target gene expression. Complementing the region-specific expression analysis, the study also found that human and murine organoids show region-specific function: the murine stomach responded to the TLR4 ligand LPS, but not the TLR2 ligand PAM3CSK4 or the TLR5 ligand flagellin, by upregulating the NF-κB target gene *Cxcl2*. Murine jejunum upregulated *Cxcl2* in response to PAM3CSK4 and flagellin, but not in response to LPS. In addition, the colon expressed *Cxcl2* in response to all of these 3 tested ligands [44]. In contrast, human organoids from all regions expressed the human *Cxcl2* analogue *IL-8* in response to flagellin, but not in response to LPS and PAM3CSK [44]. Thus, not only expression but also function of PRRs in the gut is highly organized and segment-specific. These experiments were conducted by adding the stimulus to the organoid medium, thus only stimulating the basal side, since the apical side faces the sealed-off lumen of the organoids. Other experiments demonstrated that there is an even higher level of organization, since PRR expression within a segment can be restricted to specific cell types or even subcellular locations, such as only the basal compartment. These are outlined in more detail below.

Every region of the GI tract expresses its specific set of innate immune genes. Although a generalization is difficult, the current data suggest that TLRs are expressed most highly in the stomach and colon, while the small intestine is characterized by expression of inflammasome components. Currently, the underlying molecular mechanism and the evolutionary benefit are unclear. We suspect that the physical and chemical barriers covering the epithelial layers in the different gut segments each require monitoring of specific threats. For example, the stomach and colon both have a bi-layered, thick mucus layer with the inner mucus layer firmly attached to the epithelial layer, while the small intestine is covered by a thin, viscous mucus layer [46]. The different constitutions of the mucus layers in turn are likely due to the different functions of the respective gut segments (digestion vs nutrient uptake vs water resorption). It is conceivable that the mucus thickness influences the type of PRRs necessary at the specific sites.

## Specialized cells with immune function in the epithelium

A long-standing concept in epithelial innate immunity is the existence of cell type-specific innate immune recognition. Prototypes of the specialized epithelial cells with defence function are the microfold (M) cells and the Paneth cells—recently complemented by a specialized goblet cell, the sentinel goblet cell. The existence of such specialized cells highlights the intricate and redundant systems that ensure the balance of nutrient uptake, co-existence with the microbiota, and monitoring of possible invaders. Directed differentiation of organoids now also allows the study of these cells in cell culture.

M cells are located in the follicle-associated epithelium covering the Peyer’s patches. They have a unique morphology with irregular brush border and reduced microvilli structures. Their role is to transport antigens in the gut lumen across the epithelial layer to the underlying lymphoid tissue for regulation of immune responses [47]. M cells themselves as well as the follicle-associated epithelium were shown to express several TLRs [48] (reviewed in [14]). However, M cells have been difficult to study since there are not many in the epithelial tissue and they are only found near the complex structure of Peyer’s patches [47]. Nevertheless, M cells can be generated in organoid cultures using directed differentiation. For this, receptor activator of NF-κB ligand (RANKL) is added to the medium, which upregulates the transcription factor SpiB, which is characteristic for M cell differentiation [49]. Organoids generated from mice genetically deficient for the NF-κB subunit RelB are not able to produce M cells after stimulation with RANKL, indicating that NF-κB activation is essential for development of M cells [50]. In human organoid culture, in addition to RANKL, lymphotoxin and retinoic acid are crucial for inducing differentiation towards M cells. These M cells specifically take up enteric viruses like rotavirus and reovirus, indicating correct phenocopying of the natural M cell function also in organoid cultures [51]. Future organoid work will have to continue to unravel the interconnection of epithelial NF-κB signalling, M cell development and communication with immune cells.

Paneth cells are intermingled with the intestinal stem cells at the base of the crypt and have long been considered to be the guardians of the stem cell compartment because they secrete antimicrobial peptides. For example, Paneth cells secrete alpha-defensins, a process shown to be regulated by microbial patterns and innate immune mechanisms [52, 53]. In addition, NOD2, which was first detected in the crypt region of the murine small intestine [54], regulates the secretion of several alpha-defensins by Paneth cells, which in turn leads to activation of adaptive immunity. Paneth cells also express TLR5 and Paneth cell–enriched organoids express especially high levels of TLR5. RNA sequencing after stimulation reveals that while normal small intestinal organoids only express moderate levels of the TLR5 downstream target genes in response to stimulation with flagellin, such as NF-kB-induced cytokines, organoids directed to contain high numbers of Paneth cells mount a much stronger response, indicating that Paneth cells are the main responders to flagellin in the small intestine [44, 45]. In contrast to target gene expression, the most dramatic response of Paneth cells (degranulation, extrusion and cell death) is not triggered by stimulation with TLR ligands rather requires stimulation with the professional immune cell–derived cytokine interferon-gamma [55]. These results from organoids are in agreement with the observations showing Paneth cell degranulation and extrusion after interferon-gamma stimulation in vivo [55]. This elegantly underlines the system of checks and balances in the epithelial layer.

Goblet cells are important for epithelial defence because they produce the glycosylated mucins crucial for the formation of a mucus barrier on the epithelial layer (reviewed in [56]). In mice, MUC2 deficiency results in spontaneous inflammation and increases susceptibility to infection [57, 58]. Recently, a subset of goblet cells named sentinel goblet cells was described in the mouse colon. Using tissue explants, the study identified a thickening of the mucus layer in response to exposure to TLR1/2, 4 and 5 ligands, but not to TLR9, NOD1 and NOD2 ligands [59], in congruence to a previous report of *Tlr 2*,*4* and *5* being expressed in goblet cells [60]. The authors determined that the response also depended on the presence of the *Nlrp6* inflammasome and was independent of mucosal lymphocytes using tissues from a range of knockout mice [59]. Also, a previous report had demonstrated the importance of the NLRP6 inflammasome for mucus secretion by goblet cells [61]. Imaging revealed that specific goblet cells located in the apical regions of the crypt endocytosed fluorescently tagged LPS [59]. These newly termed sentinel goblet cells not only undergo rapid degranulation and epithelial expulsion after treatment with the TLR ligands but also transmit a calcium signal to neighbouring cells via intercellular cytoplasmic bridges formed by gap junctions, likely stimulating other goblet cells to increase mucus secretion [59]. Both goblet cells and Paneth cells belong to the secretory lineage. Organoids allow directed differentiation towards both cell identities, yielding organoids heavily enriched in either goblet cells or Paneth cells [62]. Comparing transcriptomes of these skewed organoids helped identify key regulators of the differentiation pathway [63]. Further analysis of the omics data as well as functional analysis of these organoids will allow better understanding of the role of both cell types in innate immune defence.

Lastly, stem cells themselves have also been reported to express specific PRRs such as TLR4 [64, 65], which are not found on the murine small intestinal villi or Paneth cells [66]. In addition, the majority of *Nod2* expression in the murine crypt appears be restricted to stem cells [67]. Stimulation with NOD2 ligand increased survival of stem cells and formation of organoids, indicating that stimulation of PRRs may also regulate gut epithelial regeneration directly.

Without a doubt, the current efforts to generate atlases of gene expression covering every cell type in ever more detail will soon give a more complete picture of the cellular organization of innate immune signalling in the gut as well as in the entire body [68–71].

## Cell polarity and side-specific innate immune responses

Finally, it is also relevant to consider that gastrointestinal epithelial cells are highly polarized, with a specialized apical side facing the lumen of the gut with its microbiota, and a basolateral side facing the tissue. Under homeostasis, MAMPs reach the apical side only. However, when the epithelial barrier is breached, microorganisms can also challenge the basolateral side. It has thus been hypothesized that epithelial cells may selectively mount a proinflammatory response only when stimulated from the basolateral side, in order to match the threat posed by the signal. For example, TLR9 was demonstrated to induce distinct signalling pathways when stimulated from the apical or basolateral side in cancer cell lines [72] and TLR5 only induced the NF-κB response gene IL-8 when stimulated from the basal side [73].

While earlier studies using antibody labelling against TLRs have reported specific expression on one side only (reviewed in [14, 16]), analysis of TLR reporter mice using staining of an HA tag did not confirm this, but instead demonstrated TLR2, 4 and 5 receptors on both apical and basal sides of the proximal colon as well as some intracellular TLR4 [45]. These apparent differences are likely due to the different technical approaches.

Organoids now allow direct functional testing of side-specific immune responses, since the cellular polarization is retained in organoids. Under standard conditions when organoids are grown in an extracellular matrix, the apical side faces the lumen of the organoid and the basal side faces the extracellular matrix [74–76]. When grown outside an extracellular matrix, the polarity can reverse [77, 78]. When cells from organoids are seeded onto standard cell culture surfaces, such as culture dishes or transwells, the apical side faces the lumen of the well [44, 79–82].

Several studies used organoids to test the general function of particular PRRs without addressing specific differences between apical and basal stimulation. These studies included the stimulus in the medium of the organoids, which, under standard conditions, stimulates the basal side of the cell. Using this technique, upregulation of NF-κB downstream target genes was identified after basal stimulation with ligands of TLR4 in the stomach, TLR2 and 3 in the small intestine and TLR2, 3, 4 and 5 in the colon of mice [44, 45, 83] and to ligands of TLR2 and 5 in the stomach and TLR5 in the small intestine and colon of human [44]. Furthermore, basal stimulation of murine colon organoids with TLR4 agonists induced cellular differentiation, especially towards the secretory lineage [64], while NOD2 agonists induced increased survival of stem cells [67, 84], and taurine stimulated NLRP6-dependent upregulation of the inflammasome downstream target gene IL-18 [85]. In all these studies, apical stimulation was not tested.

Only few studies have addressed the side-specific functions of PRRs. It is noteworthy that contrary to the studies using polarized cancer cell lines, so far, none of the studies using organoids has identified a side-specific activation of a typical NF-κB-dependent proinflammatory gene. Transwell monolayers derived from human colon organoids express similar levels of the NF-κB target gene IL-6 when stimulated from the apical or basal side with ligands of TLR1/2, 3, 4, 5, 7/8 and 9 [82]. Murine gastric organoids also responded to an apical stimulation with the TLR4 ligand LPS in several assays, including in transwells and microinjection of LPS into the lumen of organoids [44]. Murine small intestinal epithelial cells did not express the NF-κB target gene *icam1* in response to a range of ligands, irrespective of whether they were added to intact organoids, thus stimulating the basal side, or added to single, dissociated cells, thus stimulating all sides [55].

However, looking beyond the NF-κB response, a recent study identified a side-specific function of TLR3 and its importance in viral infection [82]. Results of experiments with human colon organoid-derived monolayers showed that the expression of the critical virus-defence genes type I and type III interferon was upregulated after basal stimulation with TLR3 agonist, but not after stimulation with other TLR agonists. When infected with a virus, similarly, the interferon response of the organoids was much stronger when infected from the basal side compared to infection from the apical side. This was visible in organoid-derived monolayers as well as in organoids microinjected with the virus. The study further identified the clathrin-sorting adapter AP-1B as the molecule responsible for polarized expression of TLR3. Correspondingly, mice deficient in *Ap-1b* showed exacerbated immune responses after viral infection [82]. This confirms the polarized function of PRRs and highlights the importance of this level of regulation in further modulating pathogen recognition and defence.

So far, there is no “one size fits all” answer to the polarity question and resolving the side-specific nature of PRR signalling remains a technically challenging task for the future.

## Tolerance as a response to colonization and as a default developmental program

The mechanisms contributing to the organization of epithelial innate immunity are still unclear. The main concept in this regard has been the induction of tolerance after colonization of the sterile gut during birth, the so-called window of opportunity (reviewed in [86–88]). This concept postulates a priming period of the innate and adaptive immune system after birth, which sets the stage for immune homeostasis and subsequent host-microbial interactions.

The neonatal immune system and epithelial innate immunity are uniquely equipped to master this transition from sterility to co-existence with the microbiota. At birth, the human neonatal intestine is fully mature with intestinal villi and crypts containing Paneth cells. The murine neonatal intestinal epithelial layer is more immature and undergoes a dramatic change measurable on the transcriptome level comparing different stages of development [89] and visible in the tissue architecture and cell differentiation: The crypt-villus axis is not formed yet and cell proliferation is lower, with no cell migration or exfoliation. It does not contain mature Paneth cells; however, enterocytes produce the cathelicidin-like antimicrobial peptide (CRAMP) [90]. Paneth cells appear when crypts form 2 weeks after birth [91]. At the time of weaning, the epithelium is fully formed with crypts and villi, enterocytes, goblet cells and enteroendocrine cells; Paneth cells have taken over the antimicrobial peptide production; and goblet cells have increased the production of mucins to form the mucus layer (reviewed in [92]).

This age-dependent transition of the epithelium goes hand in hand with a gradual decrease of TLR5 expression in the small intestinal epithelium. At the same time, expression of TLR3 increases during the neonatal period. Other PRRs, such as TLR2, 4 and 9, are expressed at similar levels in neonatal and adult mice [45, 93].

The mechanisms leading to regulation of PRR expression and function after birth remain unclear. As mentioned above, several studies have proposed a contribution of the environment, in particular microbial colonization, to the regulation of PRR expression after birth [40, 41] (reviewed in [14]). However, germ-free vs specific pathogen-free mice did not show differences in TLR expression in either small intestine or colon, indicating that neither upregulation of TLR3 nor downregulation of TLR5 in this early period depends on the microbiota [45, 93]. Also, in organoids, expression of many, but not all, PRR signalling components was already defined in organoids from tissues that had never been in contact with microbial products [44]. This indicates that a large part of the organization of the innate immune signalling pathways is defined independently of contact with the microbiota and appears to be determined by default developmental processes, such as the ones outlined above, which shape the general tissue identity along the GI tract. This does not exclude a further fine-tuning of PRR expression by environmental factors during adulthood.

The importance of the timely regulation of epithelial-microbial interactions becomes clear when the immature epithelium is prematurely confronted with microbial colonization: pre-term infants are prone to the development of necrotizing enterocolitis (NEC), characterized by intestinal necrosis, systemic sepsis and multiple organ failure. Although the pathogenesis is considered multifactorial, several studies have indicated that it develops in response to an imbalance between proinflammatory signalling and repair mechanisms in the premature gut (reviewed in [94]) and a contribution of PRRs has been suggested [41, 65, 95–97]. Several studies have used human fetal organoids [24, 98, 99], normal murine organoids exposed to bacteria and hypoxia to model NEC [100], or organoids from a murine NEC model as well as from NEC patients [101]. Future studies will use these established and new organoid models to further define the contribution of the epithelium in NEC.

## Modelling epithelium-dependent aspects of IBD with organoids

Loss of intestinal epithelial barrier integrity is a defining feature of IBD, i.e. CD and UC, and seems to be caused by a multifactorial interplay of genetic predisposition, environmental factors, changes of gut microbiota and alterations of the local and systemic immune response (reviewed in [27]). Current therapeutics predominately target the (aberrant) immune responses in IBD, which are associated with high rates of non-responders and side effects (reviewed in [27]). An improved understanding of the epithelium-specific contribution to the pathophysiology of IBD is required to identify novel therapeutic targets that may also be able to directly impact intestinal epithelial barrier restoration and thus mucosal healing.

To gain insights into the epithelial pathology, several groups have established living biobanks comprised of organoids generated from individual patients with UC or CD. Although this approach is obvious, so far, only few studies have reported results from organoids derived from this group of patients [102–104]. This may be explained by the observation that organoids from patients with IBD are more difficult to generate. Our own experience is that organoids generated from CD patients grow more slowly during the first passages and some samples were lost—which was associated with a higher rate of bacterial contamination in the cultures [105].

Characterization of organoids derived from patients with IBD revealed a phenotype with decreased size and budding capacity, increased rate of cell death, luminal debris and partially inverted polarization of epithelial cells [106]. Global comparison of organoids from UC or CD patients and healthy controls showed that transcriptional and methylation differences seen in the intestinal epithelium were maintained in vitro [102, 103, 107]. Also, an earlier study from organoids generated from patients with CD suggested permanent alterations of intestinal stem cells in IBD. This was based on the observation that organoids generated from active CD lesions maintained high expression levels of intestinal epithelial stem cell markers [108]. This was to some extent confirmed in a recent study in which colon organoids derived from paediatric IBD patients showed a prolonged-expression pattern of antigen-presenting genes [109].

Focusing on changes of intestinal epithelial barrier function, including loss of tight junctions and desmosomes, which are usually found in IBD [110], it was shown that organoids from CD patients maintain this phenotype of junctional alterations under culture conditions [105]. This was especially the case when organoids were generated from sites of severe inflammation [105]. A decrease in junctional proteins could also be induced in organoids from healthy donors by application of the proinflammatory cytokine TNF-α and/or IFN-γ [106, 111]. However, the fixed pattern of changes of junctional proteins in organoids from patients with IBD was observed only on the protein level but not on the mRNA level [105].

These observations suggest that a number of changes are fixed in organoids from IBD patients. The observation that some, but not all, permanent changes are only visible on protein, but not RNA-level, suggests that there are permanent alterations in post-transcriptional modifications or protein degradation in organoids from IBD patients. This however remains to be investigated in detail.

What exactly induces the permanent alterations in expression patterns of the inflamed epithelium is unclear. An effect of the microbiota was long suspected; however, a recent study demonstrated that the effect of the microbiota on the epithelium is lost over time [112]. The study compared several mouse facilities, because the different microbiome compositions of mouse colonies have been identified as confounding factors in in vivo studies. To clarify this effect, the study compared epithelial isolates and organoid cultures from germ-free mice and two separate specific pathogen-free mouse colonies with different microbiota. While the freshly isolated epithelium showed an imprint of the microbiota exposure on RNA and protein level, this effect was lost after several weeks of culture of small intestinal organoids [112]. Also, a global comparison of gene expression of organoids generated from inflamed or non-inflamed regions of the same IBD patients demonstrated that the IBD organoids of the inflamed regions lost the inflammatory gene expression already after a few weeks in culture. The transcriptomes of the organoids were then clustered per patient; thus, the permanent differences between IBD and healthy controls remained. The inflammatory phenotype in the IBD organoids could then be re-induced by addition of a cytokine cocktail [107]. Overall, this suggests that permanent changes observed in organoids from IBD patients are independent of contact with microbiota or cytokines.

Thus, although it is reasonable to speculate that some of the permanent changes in intestinal epithelial cells from patients with IBD may be caused by changes in innate immune signalling, the evidence for this is currently scarce. In addition, not all of the epithelial changes observed in the inflamed regions in the gut are permanently conserved in the purified epithelium, indicating important contributions from the local environment. In the future, new studies with more complex organoid models, also incorporating immune cells, inflammatory cytokine stimulation and co-cultures with microorganisms, should help to address this hypothesis in more detail.

## Conclusions, future perspectives and outlook

In summary, while many diseases within the GI tract remain incompletely understood, increasing evidence points to a critical role of the gastrointestinal epithelium in the pathogenesis of many of them—although its specific role remains unclear.

The limited knowledge of innate immune function of the gastrointestinal epithelium has been attributed to a lack of appropriate experimental models. With the implementation of the organoid technology, a major step has been taken to overcome this problem. Organoids generated from each region of the gastrointestinal tract will add significantly to the existing knowledge. As revealed by previous studies, the generation of organoids provides the crucial advantage of being able to observe responses of primary gastrointestinal cells as opposed to transformed cell lines, which are mostly generated from gastrointestinal malignant tumours. One of the most fascinating and interesting features of organoids is that they maintain specific characteristics of the segment of the gastrointestinal tract they were generated from as part of their cell identity during adulthood. According to current experimental studies, the regional identity is fixed in the gastrointestinal stem cells. In this context, a remaining issue will be to determine exactly how and when during development this intrinsic programming occurs.

**Fig. 4:**
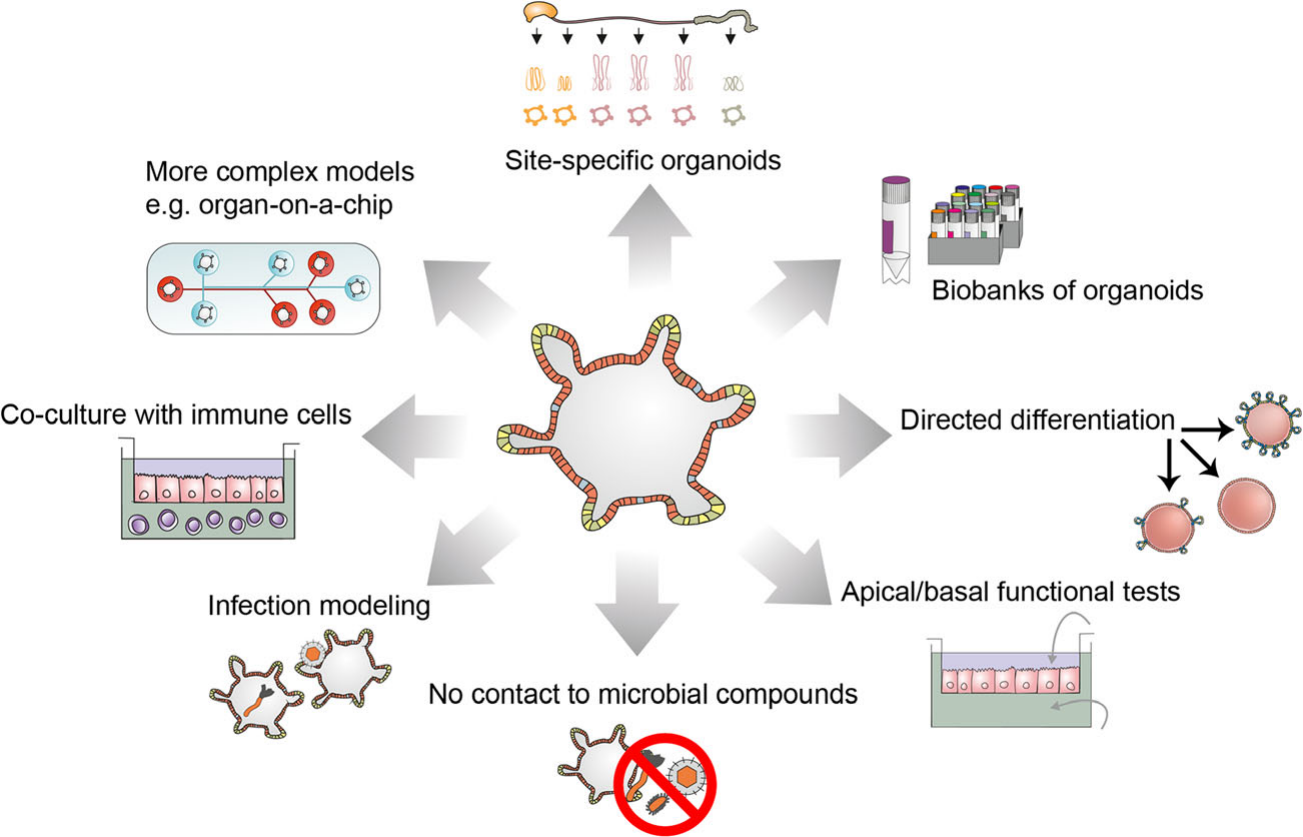
The use of organoids for the study of epithelial innate immunity. Clockwise: Organoids have been generated from different segments of the gastrointestinal tract and have been shown to retain tissue identity. Organoids from patients can be stored in biobanks to enable research on specific pathologies such as IBD. Using culture modifications, the cells in organoids can also be directed towards specific cell identities, such as secretory cells, enterocytes and M cells. Because organoids retain the polarity of the cells, they also allow testing of apical and basal stimulation. For this, cells can be seeded in transwell systems. Organoids are a reductionist model, which is not in contact with microbial compounds under standard conditions but can be used to study infection with bacteria and viruses and to study interaction with immune cells in co-culture experiments. In the future, more complex models will also allow combinations of several organoid types in microfluidic lab-on-a-chip devices

When looking at specific concepts of how the epithelium may respond to or interact with the environment, organoid technology has now enabled illumination of the differential and segment-specific expression and function of PRRs within the gastrointestinal epithelium. The overall functional consequences for the complex regulatory systems within the whole gastrointestinal tract are still unclear and will have to be addressed in the future. For this, it will also be important to co-culture organoids with immune cells (reviewed in [113]), cells of the enteric nervous system [114] and luminal factors such as bacterial co-cultures (reviewed in [115]) (Fig. 4).

A further important aspect is that organoids generated from patient tissue affected by GI diseases such as IBD maintain some of the characteristics seen in the corresponding tissue specimens they were derived from. This offers the unique possibility to further unravel the epithelium- and disease-specific contribution to the pathogenesis of GI diseases—not only those involving inflammation-induced changes but also those involving changes in malignant diseases. Both of them may turn out to involve a specific contribution of epithelium-derived innate immunity. For this, the systematic establishment of “living biobanks” would be an important step. As a vision for the future, such living biobanks could be attached to already existing biobanks, which currently provide solely “dead” biomaterials. This would represent another important step not only for research but also in facilitating individualized diagnostics and therapy for patients.

Box 1. Innate immunity and epithelial cells in the GI tract

**Table:** 

In homeostasis, it is likely that due to the mucus layer, which poses a diffusion barrier, only low concentrations of MAMPs reach the epithelial layer [59]. At these low, MAMPs seem not to elicit a proinflammatory response but still impact the epithelial cells in several ways. Mice deficient for either TLR2, 4, 5, and 9, the TLR-pathway mediator MyD88 or the NLRP6 inflammasome are highly susceptible to experimentally induced colitis [116–119]. Mice devoid of TLR2 or MyD88 show disrupted tight junctions, more apoptotic epithelial cells and higher barrier permeability [116]. NLRP6 deficiency leads to reduced mucus secretion [61]. Mice devoid of NOD2 show higher epithelial cell death rates after treatment with doxorubicin, indicating that they are more susceptible [67]. In line with this observation, patients with mutations in the NOD2 gene are predisposed to develop IBDs especially CD [120]. While these studies clearly point to the importance of innate immune signalling for epithelial homeostasis, it is unclear whether the observed impact is due to the innate immune signalling in epithelial cells or in professional immune cells. To disentangle the interplay of professional immune cells and epithelial cells, several studies have used epithelium-specific knockouts, or more recently, epithelial organoids. In mouse models, none of the epithelial-specific deletions of PRRs leads to spontaneous inflammation. However, mice with epithelial-specific knockout of MyD88 are more susceptible to experimental colitis and show severe barrier disruption, impaired goblet and Paneth cell responses [121] and reduced production of mucin and antimicrobial peptides [121, 122]. Small intestinal organoids do not mount an inflammatory response to several purified PRR ligands [55], although this cannot be generalized and depends on the species, location and age of the tissue the organoids are generated from [44, 45]. The absence of a spontaneous inflammatory phenotype in epithelial cell-specific PRR knockout models does support the hypothesis that factors other than a general inflammatory response of the epithelium have an impact on epithelial homeostasis. For example, stimulation with the NOD2 agonist muramyl dipeptide (MDP) increased the number of organoids growing out of isolated stem cells, indicating that the innate immune signalling supported survival of the stem cells [67, 84]. Furthermore, data from mice highlight the importance of the anti-apoptotic effects of NF-κB signalling in response to other stimuli, such as TNF-α [123]. Interestingly, in humans, polymorphisms in innate immune genes including *NOD2* and *TLR4* are associated with an increased risk to develop IBD [124] and blockage of TNF-α is currently the most efficacious treatment for IBD in some patients (reviewed in [125]). A picture emerges, in which a low level of innate immune stimulation is important for mucus secretion, barrier integrity and epithelial cell survival. Its impairment may allow translocation of intestinal bacteria from the lumen into the subepithelial tissue, leading to inflammation.	

## References

[1] Goodwin CS (1984) Microbes and infections of the gut

[2] Sekirov I, Russell SL, Antunes LCM, Finlay BB (2010). Gut microbiota in health and disease. Physiol Rev.

[3] Simon GL, Gorbach SL (1986). The human intestinal microflora. Dig Dis Sci.

[4] Bartfeld S, Clevers H (2017) Stem cell-derived organoids and their application for medical research and patient treatment. J Mol Med:1–10. 10.1007/s00109-017-1531-7 10.1007/s00109-017-1531-7 28391362

[5] Barker N (2014). Adult intestinal stem cells: critical drivers of epithelial homeostasis and regeneration. Nat Rev Mol Cell Biol.

[6] Bartfeld S, Koo B-K (2017). Adult gastric stem cells and their niches. Wiley Interdiscip Rev Dev Biol.

[7] Liwinski T, Zheng D, Elinav E (2020). The microbiome and cytosolic innate immune receptors. Immunol Rev.

[8] Burgueño JF, Abreu MT (2020). Epithelial Toll-like receptors and their role in gut homeostasis and disease. Nat Rev Gastroenterol Hepatol.

[9] Christgen S, Kanneganti T-D (2020). Inflammasomes and the fine line between defense and disease. Curr Opin Immunol.

[10] Ying L, Ferrero RL (2019) Role of NOD1 and ALPK1/TIFA signalling in innate immunity against Helicobacter pylori. Infection pp:159–177 10.1007/978-3-030-15138-6_7 31123889

[11] Takeuchi O, Akira S (2010). Pattern recognition receptors and inflammation. Cell.

[12] Peterson LW, Artis D (2014). Intestinal epithelial cells: regulators of barrier function and immune homeostasis. Nat Rev Immunol.

[13] Pott J, Hornef M (2012). Innate immune signalling at the intestinal epithelium in homeostasis and disease. EMBO Rep.

[14] Abreu MT (2010). Toll-like receptor signalling in the intestinal epithelium: how bacterial recognition shapes intestinal function. Nat Rev Immunol.

[15] Zhang K, Hornef MW, Dupont A (2015). The intestinal epithelium as guardian of gut barrier integrity. Cell Microbiol.

[16] Yu S, Gao N (2015). Compartmentalizing intestinal epithelial cell toll-like receptors for immune surveillance. Cell Mol Life Sci.

[17] Zhou P, She Y, Dong N, Li P, He H, Borio A, Wu Q, Lu S, Ding X, Cao Y, Xu Y, Gao W, Dong M, Ding J, Wang DC, Zamyatina A, Shao F (2018). Alpha-kinase 1 is a cytosolic innate immune receptor for bacterial ADP-heptose. Nature..

[18] Milivojevic M, Dangeard A-S, Kasper CA, Tschon T, Emmenlauer M, Pique C, Schnupf P, Guignot J, Arrieumerlou C (2017). ALPK1 controls TIFA/TRAF6-dependent innate immunity against heptose-1,7-bisphosphate of gram-negative bacteria. PLoS Pathog.

[19] Zimmermann S, Pfannkuch L, Al-Zeer MA (2017). ALPK1- and TIFA-dependent innate immune response triggered by the helicobacter pylori type IV secretion system. Cell Rep.

[20] Abreu MT, Fukata M, Arditi M (2005). TLR signaling in the gut in health and disease. J Immunol.

[21] Pédron T, Sansonetti P (2008). Commensals, bacterial pathogens and intestinal inflammation: an intriguing Ménage à Trois. Cell Host and Microbe.

[22] Clevers H (2013). The intestinal crypt, a prototype stem cell compartment. Cell.

[23] Lancaster M, Takebe T, Lancaster M (2017). Advances in organoid technology: Hans Clevers, Madeline Lancaster, and Takanori Takebe. Cell Stem Cell.

[24] Kraiczy J, Nayak KM, Howell KJ (2017). DNA methylation defines regional identity of human intestinal epithelial organoids and undergoes dynamic changes during development. Gut gutjnl.

[25] Elmentaite R, Ross ADB, Roberts K, James KR, Ortmann D, Gomes T, Nayak K, Tuck L, Pritchard S, Bayraktar OA, Heuschkel R, Vallier L, Teichmann SA, Zilbauer M (2020) Single-cell sequencing of developing human gut reveals transcriptional links to childhood Crohn’s disease. Dev Cell 0: , 55, 771, 783.e5 10.1016/j.devcel.2020.11.010 PMC7762816 33290721

[26] Podolsky DK (2002). Inflammatory bowel disease. N Engl J Med.

[27] Schlegel N, Boerner K, Waschke J (2020) Targeting desmosomal adhesion and signalling for intestinal barrier stabilization in inflammatory bowel diseases-Lessons from experimental models and patients. Acta Physiol (Oxf):e13492. 10.1111/apha.13492 10.1111/apha.13492 32419327

[28] Kavanagh ME, O’Sullivan KE, O’Hanlon C, O’Sullivan JN, Lysaght J, Reynolds JV (2014). The esophagitis to adenocarcinoma sequence; the role of inflammation. Cancer Lett.

[29] Grivennikov SI, Greten FR, Karin M (2010). Immunity, inflammation, and cancer. Cell.

[30] Barsouk A, Rawla P, Barsouk A, Thandra KC (2019) Epidemiology of cancers of the small intestine: trends, risk factors, and prevention. Med Sci (Basel) 7. 10.3390/medsci7030046 10.3390/medsci7030046 PMC6473503 30884915

[31] Karam SM (1999). Lineage commitment and maturation of epithelial cells in the gut. Front Biosci.

[32] Johansson MEV, Sjövall H, Hansson GC (2013). The gastrointestinal mucus system in health and disease. Nat Rev Gastroenterol Hepatol.

[33] Koelz HR (1992). Gastric acid in vertebrates. Scand J Gastroenterol.

[34] Cheng H, Leblond CP (1974). Origin, differentiation and renewal of the four main epithelial cell types in the mouse small intestine I. Columnar cell. American Journal of Anatomy.

[35] Johansson MEV, Ambort D, Pelaseyed T, Schütte A, Gustafsson JK, Ermund A, Subramani DB, Holmén-Larsson JM, Thomsson KA, Bergström JH, van der Post S, Rodriguez-Piñeiro AM, Sjövall H, Bäckström M, Hansson GC (2011). Composition and functional role of the mucus layers in the intestine. Cell Mol Life Sci.

[36] Osuntokun B, Kocoshis SA (2006) Anatomy and physiology of the small and large intestine, Third Edit. Elsevier Inc.

[37] Chang WWL, Leblond CP (1971). Renewal of the epithelium in the descending colon of the mouse. I. Presence of three cell populations: vacuolated-columnar, mucous and argentaffin. American Journal of Anatomy.

[38] Mowat AM, Agace WW (2014). Regional specialization within the intestinal immune system. Nat Rev Immunol.

[39] Shaw-Smith CJ, Walters JR (1997). Regional expression of intestinal genes for nutrient absorption. Gut.

[40] Lotz M, Gütle D, Walther S (2006). Postnatal acquisition of endotoxin tolerance in intestinal epithelial cells. J Cell Biol.

[41] Gribar SC, Sodhi CP, Richardson WM (2009). Reciprocal expression and signaling of TLR4 and TLR9 in the pathogenesis and treatment of necrotizing enterocolitis. Journal of immunology (Baltimore, Md : 1950).

[42] Bäckhed F, Hornef M (2003). Toll-like receptor 4-mediated signaling by epithelial surfaces: necessity or threat?. Microbes Infect.

[43] Ortega-Cava CF, Ishihara S, Rumi MAK (2003). Strategic compartmentalization of Toll-like receptor 4 in the mouse gut. Journal of immunology (Baltimore, Md : 1950).

[44] Kayisoglu O, Weiss F, Niklas C (2020). Location-specific cell identity rather than exposure to GI microbiota defines many innate immune signalling cascades in the gut epithelium. Gut gutjnl.

[45] Price AE, Shamardani K, Lugo KA (2018). A map of toll-like receptor expression in the intestinal epithelium reveals distinct spatial, cell type-specific, and temporal patterns. Immunity.

[46] Ermund A, Schütte A, Johansson MEV, Gustafsson JK, Hansson GC (2013). Studies of mucus in mouse stomach, small intestine, and colon. I. Gastrointestinal mucus layers have different properties depending on location as well as over the Peyer’s patches. Am J Physiol Gastrointest Liver Physiol.

[47] Mabbott NA, Donaldson DS, Ohno H, Williams IR, Mahajan A (2013). Microfold (M) cells: important immunosurveillance posts in the intestinal epithelium. Mucosal Immunol.

[48] Cashman SB, Morgan JG (2009). Transcriptional analysis of Toll-like receptors expression in M cells. Mol Immunol.

[49] de Lau W, Kujala P, Schneeberger K, Middendorp S, Li VSW, Barker N, Martens A, Hofhuis F, DeKoter RP, Peters PJ, Nieuwenhuis E, Clevers H (2012). Peyer’s patch M cells derived from Lgr5+ stem cells require SpiB and are induced by RankL in cultured “Miniguts”. Mol Cell Biol.

[50] Kanaya T, Sakakibara S, Jinnohara T, Hachisuka M, Tachibana N, Hidano S, Kobayashi T, Kimura S, Iwanaga T, Nakagawa T, Katsuno T, Kato N, Akiyama T, Sato T, Williams IR, Ohno H (2018). Development of intestinal M cells and follicle-associated epithelium is regulated by TRAF6-mediated NF-κB signaling. J Exp Med.

[51] Ding S, Song Y, Brulois KF, Pan J, Co JY, Ren L, Feng N, Yasukawa LL, Sánchez-Tacuba L, Wosen JE, Mellins ED, Monack DM, Amieva MR, Kuo CJ, Butcher EC, Greenberg HB (2020). Retinoic acid and lymphotoxin signaling promote differentiation of human intestinal M cells. Gastroenterology..

[52] Ayabe T, Satchell DP, Wilson CL, Parks WC, Selsted ME, Ouellette AJ (2000). Secretion of microbicidal α-defensins by intestinal Paneth cells in response to bacteria. Nat Immunol.

[53] Vaishnava S, Behrendt CL, Ismail AS, Eckmann L, Hooper LV (2008). Paneth cells directly sense gut commensals and maintain homeostasis at the intestinal host-microbial interface. Proc Natl Acad Sci.

[54] Kobayashi KS, Chamaillard M, Ogura Y, Henegariu O, Inohara N, Nuñez G, Flavell RA (2005). Nod2-dependent regulation of innate and adaptive immunity in the intestinal tract. Science.

[55] Farin HF, Karthaus WR, Kujala P, Rakhshandehroo M, Schwank G, Vries RGJ, Kalkhoven E, Nieuwenhuis EES, Clevers H (2014). Paneth cell extrusion and release of antimicrobial products is directly controlled by immune cell-derived IFN-γ. J Exp Med.

[56] Knoop KA, Newberry RD (2018). Goblet cells: multifaceted players in immunity at mucosal surfaces. Mucosal Immunol.

[57] Van der Sluis M, De Koning BAE, De Bruijn ACJM (2006). Muc2-deficient mice spontaneously develop colitis, indicating that MUC2 is critical for colonic protection. Gastroenterology.

[58] Bergstrom KSBB, Kissoon-Singh V, Gibson DL (2010). Muc2 protects against lethal infectious colitis by disassociating pathogenic and commensal bacteria from the colonic mucosa. PLoS Pathog.

[59] Birchenough GMH, Nystrom EEL, Johansson MEV, Hansson GC (2016). A sentinel goblet cell guards the colonic crypt by triggering Nlrp6-dependent Muc2 secretion. Science.

[60] Knoop KA, McDonald KG, McCrate S (2015). Microbial sensing by goblet cells controls immune surveillance of luminal antigens in the colon. Mucosal Immunol.

[61] Wlodarska M, Thaiss CA, Nowarski R, Henao-Mejia J, Zhang JP, Brown EM, Frankel G, Levy M, Katz MN, Philbrick WM, Elinav E, Finlay BB, Flavell RA (2014). NLRP6 inflammasome orchestrates the colonic host-microbial interface by regulating goblet cell mucus secretion. Cell.

[62] Yin X, Farin HF, Van Es JH (2014). Niche-independent high-purity cultures of Lgr5 + intestinal stem cells and their progeny. Nat Methods.

[63] Treveil A, Sudhakar P, Matthews ZJ, Wrzesiński T, Jones EJ, Brooks J, Ölbei M, Hautefort I, Hall LJ, Carding SR, Mayer U, Powell PP, Wileman T, di Palma F, Haerty W, Korcsmáros T (2020). Regulatory network analysis of Paneth cell and goblet cell enriched gut organoids using transcriptomics approaches. Molecular Omics.

[64] Naito T, Mulet C, De Castro C (2017). Lipopolysaccharide from crypt-specific core microbiota modulates the colonic epithelial proliferation-to-differentiation balance. mBio.

[65] Neal MD, Sodhi CP, Jia H, Dyer M, Egan CE, Yazji I, Good M, Afrazi A, Marino R, Slagle D, Ma C, Branca MF, Prindle T, Grant Z, Ozolek J, Hackam DJ (2012). Toll-like receptor 4 is expressed on intestinal stem cells and regulates their proliferation and apoptosis via the p53 up-regulated modulator of apoptosis. J Biol Chem.

[66] Tanabe H, Ayabe T, Bainbridge B, Guina T, Ernst RK, Darveau RP, Miller SI, Ouellette AJ (2005). Mouse Paneth cell secretory responses to cell surface glycolipids of virulent and attenuated pathogenic bacteria. Infect Immun.

[67] Nigro G, Rossi R, Commere PH, Jay P, Sansonetti PJ (2014). The cytosolic bacterial peptidoglycan sensor Nod2 affords stem cell protection and links microbes to gut epithelial regeneration. Cell Host and Microbe.

[68] Chen J, Lau BT, Andor N, Grimes SM, Handy C, Wood-Bouwens C, Ji HP (2019). Single-cell transcriptome analysis identifies distinct cell types and niche signaling in a primary gastric organoid model. Sci Rep.

[69] Grün D, Lyubimova A, Kester L, Wiebrands K, Basak O, Sasaki N, Clevers H, van Oudenaarden A (2015). Single-cell messenger RNA sequencing reveals rare intestinal cell types. Nature.

[70] Haber AL, Biton M, Rogel N, Herbst RH, Shekhar K, Smillie C, Burgin G, Delorey TM, Howitt MR, Katz Y, Tirosh I, Beyaz S, Dionne D, Zhang M, Raychowdhury R, Garrett WS, Rozenblatt-Rosen O, Shi HN, Yilmaz O, Xavier RJ, Regev A (2017). A single-cell survey of the small intestinal epithelium. Nature.

[71] Schaum N, Karkanias J, Neff NF (2018). Single-cell transcriptomics of 20 mouse organs creates a Tabula Muris. Nature.

[72] Lee J, Mo JH, Katakura K, Alkalay I, Rucker AN, Liu YT, Lee HK, Shen C, Cojocaru G, Shenouda S, Kagnoff M, Eckmann L, Ben-Neriah Y, Raz E (2006). Maintenance of colonic homeostasis by distinctive apical TLR9 signalling in intestinal epithelial cells. Nat Cell Biol.

[73] Gewirtz AT, Navas TA, Lyons S, Godowski PJ, Madara JL (2001). Cutting edge: bacterial flagellin activates basolaterally expressed TLR5 to induce epithelial proinflammatory gene expression. J Immunol.

[74] Bartfeld S, Bayram T, Van De Wetering M (2015). In vitro expansion of human gastric epithelial stem cells and their responses to bacterial infection. Gastroenterology.

[75] Sato T, Vries RG, Snippert HJ, van de Wetering M, Barker N, Stange DE, van Es JH, Abo A, Kujala P, Peters PJ, Clevers H (2009). Single Lgr5 stem cells build crypt-villus structures in vitro without a mesenchymal niche. Nature.

[76] Sato T, Stange DE, Ferrante M, Vries RGJ, van Es JH, van den Brink S, van Houdt WJ, Pronk A, van Gorp J, Siersema PD, Clevers H (2011). Long-term expansion of epithelial organoids from human colon, adenoma, adenocarcinoma, and Barrett’s epithelium. Gastroenterology.

[77] Co JY, Margalef-Català M, Li X (2019). Controlling epithelial polarity: a human enteroid model for host-pathogen interactions. Cell Reports.

[78] Huang JY, Sweeney EG, Sigal M, Zhang HC, Remington SJ, Cantrell MA, Kuo CJ, Guillemin K, Amieva MR (2015). Chemodetection and destruction of host urea allows Helicobacter pylori to locate the epithelium. Cell Host and Microbe.

[79] Bartfeld S, Clevers H (2015) Organoids as model for infectious diseases: culture of human and murine stomach organoids and microinjection of helicobacter pylori. Journal of Visualized Experiments:1–9. 10.3791/53359 10.3791/53359 PMC4692704 26650279

[80] Boccellato F, Woelffling S, Imai-Matsushima A, Sanchez G, Goosmann C, Schmid M, Berger H, Morey P, Denecke C, Ordemann J, Meyer TF (2019). Polarised epithelial monolayers of the gastric mucosa reveal insights into mucosal homeostasis and defence against infection. Gut.

[81] Schlaermann P, Toelle B, Berger H, Schmidt SC, Glanemann M, Ordemann J, Bartfeld S, Mollenkopf HJ, Meyer TF (2016). A novel human gastric primary cell culture system for modelling Helicobacter pylori infection in vitro. Gut.

[82] Stanifer ML, Mukenhirn M, Muenchau S, Pervolaraki K, Kanaya T, Albrecht D, Odendall C, Hielscher T, Haucke V, Kagan JC, Bartfeld S, Ohno H, Boulant S (2020). Asymmetric distribution of TLR3 leads to a polarized immune response in human intestinal epithelial cells. Nat Microbiol.

[83] Davies JM, Santaolalla R, Von Furstenberg RJ (2015). The viral mimetic polyinosinic: polycytidylic acid alters the growth characteristics of small intestinal and colonic crypt cultures. PLoS One.

[84] Levy A, Stedman A, Deutsch E, Donnadieu F, Virgin HW, Sansonetti PJ, Nigro G (2020). Innate immune receptor NOD2 mediates LGR5+ intestinal stem cell protection against ROS cytotoxicity via mitophagy stimulation. Proc Natl Acad Sci U S A.

[85] Levy M, Thaiss CA, Zeevi D, Dohnalová L, Zilberman-Schapira G, Mahdi JA, David E, Savidor A, Korem T, Herzig Y, Pevsner-Fischer M, Shapiro H, Christ A, Harmelin A, Halpern Z, Latz E, Flavell RA, Amit I, Segal E, Elinav E (2015). Microbiota-modulated metabolites shape the intestinal microenvironment by regulating NLRP6 inflammasome signaling. Cell.

[86] Renz H, Adkins BD, Bartfeld S, Blumberg RS, Farber DL, Garssen J, Ghazal P, Hackam DJ, Marsland BJ, McCoy KD, Penders J, Prinz I, Verhasselt V, von Mutius E, Weiser JN, Wesemann DR, Hornef MW (2018). The neonatal window of opportunity-early priming for life. J Allergy Clin Immunol.

[87] Hornef MW, Torow N (2020). ‘Layered immunity’ and the ‘neonatal window of opportunity’ – timed succession of non-redundant phases to establish mucosal host–microbial homeostasis after birth. Immunology.

[88] Torow N, Hornef MW (2017). The neonatal window of opportunity: setting the stage for life-long host-microbial interaction and immune homeostasis. J Immunol.

[89] Rakoff-Nahoum S, Kong Y, Kleinstein SH, Subramanian S, Ahern PP, Gordon JI, Medzhitov R (2015). Analysis of gene–environment interactions in postnatal development of the mammalian intestine. Proc Natl Acad Sci.

[90] Ménard S, Förster V, Lotz M (2008). Developmental switch of intestinal antimicrobial peptide expression. J Exp Med.

[91] Bry L, Falk P, Huttner K, Ouellette A, Midtvedt T, Gordon JI (1994). Paneth cell differentiation in the developing intestine of normal and transgenic mice. Proc Natl Acad Sci.

[92] De Santa BP, Van Den Brink GR, Roberts DJ (2003). Development and differentiation of the intestinal epithelium. Cell Mol Life Sci.

[93] Pott J, Stockinger S, Torow N, Smoczek A, Lindner C, McInerney G, Bäckhed F, Baumann U, Pabst O, Bleich A, Hornef MW (2012). Age-dependent TLR3 expression of the intestinal epithelium contributes to rotavirus susceptibility. PLoS Pathog.

[94] Hackam DJ, Sodhi CP (2018). Toll-like receptor–mediated intestinal inflammatory imbalance in the pathogenesis of necrotizing enterocolitis. Cmgh.

[95] Afrazi A, Branca MF, Sodhi CP, Good M, Yamaguchi Y, Egan CE, Lu P, Jia H, Shaffiey S, Lin J, Ma C, Vincent G, Thomas P, Weyandt S, Neal MD, Ozolek JA, Wiersch J, Tschurtschenthaler M, Shiota C, Gittes GK, Billiar TR, Mollen K, Kaser A, Blumberg R, Hackam DJ (2014). Toll-like receptor 4-mediated endoplasmic reticulum stress in intestinal crypts induces necrotizing enterocolitis. J Biol Chem.

[96] Richardson WM, Sodhi CP, Russo A (2010). Nucleotide-binding oligomerization domain-2 inhibits toll-like receptor-4 signaling in the intestinal epithelium. Gastroenterology.

[97] Sodhi CP, Shi X-H, Richardson WM (2010). Toll-like receptor-4 inhibits enterocyte proliferation via impaired β-catenin signaling in necrotizing enterocolitis. YGAST.

[98] Senger S, Ingano L, Freire R et al (2018) Human fetal-derived enterospheres provide insights on intestinal development and a novel model to study necrotizing enterocolitis (NEC). Cellular and Molecular gastroenterology and Hepatology:1–20. 10.1016/j.jcmgh.2018.01.014 10.1016/j.jcmgh.2018.01.014 PMC6009798 29930978

[99] Roodsant T, Navis M, Aknouch I, Renes IB, van Elburg RM, Pajkrt D, Wolthers KC, Schultsz C, van der Ark KCH, Sridhar A, Muncan V (2020). A human 2D primary organoid-derived epithelial monolayer model to study host-pathogen interaction in the small intestine. Front Cell Infect Microbiol.

[100] Werts AD, Fulton WB, Ladd MR, Saad-Eldin A, Chen YX, Kovler ML, Jia H, Banfield EC, Buck RH, Goehring K, Prindle T, Wang S, Zhou Q, Lu P, Yamaguchi Y, Sodhi CP, Hackam DJ (2020). A novel role for necroptosis in the pathogenesis of necrotizing enterocolitis. Cell Mol Gastroenterol Hepatol.

[101] Li B, Lee C, Cadete M, Zhu H, Koike Y, Hock A, Wu RY, Botts SR, Minich A, Alganabi M, Chi L, Zani-Ruttenstock E, Miyake H, Chen Y, Mutanen A, Ngan B, Johnson-Henry KC, de Coppi P, Eaton S, Määttänen P, Delgado-Olguin P, Sherman PM, Zani A, Pierro A (2019). Impaired Wnt/β-catenin pathway leads to dysfunction of intestinal regeneration during necrotizing enterocolitis. Cell Death and Disease.

[102] Dotti I, Mora-Buch R, Ferrer-Picón E, Planell N, Jung P, Masamunt MC, Leal RF, Martín de Carpi J, Llach J, Ordás I, Batlle E, Panés J, Salas A (2017). Alterations in the epithelial stem cell compartment could contribute to permanent changes in the mucosa of patients with ulcerative colitis. Gut.

[103] Howell KJ, Kraiczy J, Nayak KM, Gasparetto M, Ross A, Lee C, Mak TN, Koo BK, Kumar N, Lawley T, Sinha A, Rosenstiel P, Heuschkel R, Stegle O, Zilbauer M (2018). DNA methylation and transcription patterns in intestinal epithelial cells from pediatric patients with inflammatory bowel diseases differentiate disease subtypes and associate with outcome. Gastroenterology.

[104] Noben M, Verstockt B, De Bruyn M (2017). Epithelial organoid cultures from patients with ulcerative colitis and Crohn’s disease: a truly long-term model to study the molecular basis for inflammatory bowel disease?. Gut.

[105] Meir M, Salm J, Fey C, Schweinlin M, Kollmann C, Kannapin F, Germer CT, Waschke J, Beck C, Burkard N, Metzger M, Schlegel N (2020). Enteroids generated from patients with severe inflammation in Crohn’s disease maintain alterations of junctional proteins. J Crohns Colitis.

[106] d’Aldebert E, Quaranta M, Sébert M, Bonnet D, Kirzin S, Portier G, Duffas JP, Chabot S, Lluel P, Allart S, Ferrand A, Alric L, Racaud-Sultan C, Mas E, Deraison C, Vergnolle N (2020) Characterization of human Colon organoids from inflammatory bowel disease patients. Front Cell Dev Biol 8. 10.3389/fcell.2020.00363 10.3389/fcell.2020.00363 PMC7287042 32582690

[107] Arnauts K, Verstockt B, Ramalho AS, Vermeire S, Verfaillie C, Ferrante M (2020). Ex vivo mimicking of inflammation in organoids derived from patients with ulcerative colitis. Gastroenterology.

[108] Suzuki K, Murano T, Shimizu H, Ito G, Nakata T, Fujii S, Ishibashi F, Kawamoto A, Anzai S, Kuno R, Kuwabara K, Takahashi J, Hama M, Nagata S, Hiraguri Y, Takenaka K, Yui S, Tsuchiya K, Nakamura T, Ohtsuka K, Watanabe M, Okamoto R (2018). Single cell analysis of Crohn’s disease patient-derived small intestinal organoids reveals disease activity-dependent modification of stem cell properties. J Gastroenterol.

[109] Kelsen JR, Dawany N, Conrad MA, Karakasheva TA, Maurer K, Wei JM, Uman S, Dent MH, Behera R, Bryant LM, Ma X, Moreira L, Chatterji P, Shraim R, Merz A, Mizuno R, Simon LA, Muir AB, Giraudo C, Behrens EM, Whelan KA, Devoto M, Russo PA, Andres SF, Sullivan KE, Hamilton KE (2020). Colonoids from patients with pediatric inflammatory bowel disease exhibit decreased growth associated with inflammation severity and durable upregulation of antigen presentation genes. Inflamm Bowel Dis.

[110] Spindler V, Meir M, Vigh B, Flemming S, Hütz K, Germer CT, Waschke J, Schlegel N (2015). Loss of Desmoglein 2 contributes to the pathogenesis of Crohn’s disease. Inflamm Bowel Dis.

[111] Meir M, Burkard N, Ungewiß H, Diefenbacher M, Flemming S, Kannapin F, Germer CT, Schweinlin M, Metzger M, Waschke J, Schlegel N (2019). Neurotrophic factor GDNF regulates intestinal barrier function in inflammatory bowel disease. J Clin Invest.

[112] Hausmann A, Russo G, Grossmann J, Zünd M, Schwank G, Aebersold R, Liu Y, Sellin ME, Hardt WD (2020). Germ-free and microbiota-associated mice yield small intestinal epithelial organoids with equivalent and robust transcriptome/proteome expression phenotypes. Cell Microbiol.

[113] Bar-Ephraim YE, Kretzschmar K, Clevers H (2020). Organoids in immunological research. Nat Rev Immunol.

[114] Workman MJ, Mahe MM, Trisno S, Poling HM, Watson CL, Sundaram N, Chang CF, Schiesser J, Aubert P, Stanley EG, Elefanty AG, Miyaoka Y, Mandegar MA, Conklin BR, Neunlist M, Brugmann SA, Helmrath MA, Wells JM (2017). Engineered human pluripotent-stem-cell-derived intestinal tissues with a functional enteric nervous system. Nat Med.

[115] Pompaiah M, Bartfeld S (2017). Gastric organoids: an emerging model system to study helicobacter pylori pathogenesis.

[116] Cario E, Gerken G, Podolsky DK (2007). Toll-like receptor 2 controls mucosal inflammation by regulating epithelial barrier function. Gastroenterology.

[117] Elinav E, Strowig T, Kau AL, Henao-Mejia J, Thaiss CA, Booth CJ, Peaper DR, Bertin J, Eisenbarth SC, Gordon JI, Flavell RA (2011). NLRP6 inflammasome regulates colonic microbial ecology and risk for colitis. Cell.

[118] Rakoff-Nahoum S, Paglino J, Eslami-Varzaneh F, Edberg S, Medzhitov R (2004). Recognition of commensal microflora by Toll-like receptors is required for intestinal homeostasis. Cell.

[119] Rhee SH, Im E, Riegler M, Kokkotou E, O’Brien M, Pothoulakis C (2005). Pathophysiological role of Toll-like receptor 5 engagement by bacterial flagellin in colonic inflammation. Proc Natl Acad Sci.

[120] Hugot J, Chamaillard M, Zouali H, et al (2001) Association of NOD2 leucine-rich repeat variants with susceptibility to Crohn’ s disease. 599–603 10.1038/35079107 11385576

[121] Bhinder G, Stahl M, Sham HP, Crowley SM, Morampudi V, Dalwadi U, Ma C, Jacobson K, Vallance BA (2014). Intestinal epithelium-specific MyD88 signaling impacts host susceptibility to infectious colitis by promoting protective goblet cell and antimicrobial responses. Infect Immun.

[122] Frantz AL, Rogier EW, Weber CR, Shen L, Cohen DA, Fenton LA, Bruno MEC, Kaetzel CS (2012). Targeted deletion of MyD88 in intestinal epithelial cells results in compromised antibacterial immunity associated with downregulation of polymeric immunoglobulin receptor, mucin-2, and antibacterial peptides. Mucosal Immunol.

[123] Nenci A, Becker C, Wullaert A, Gareus R, van Loo G, Danese S, Huth M, Nikolaev A, Neufert C, Madison B, Gumucio D, Neurath MF, Pasparakis M (2007). Epithelial NEMO links innate immunity to chronic intestinal inflammation. Nature.

[124] Gaya DR, Russell RK, Nimmo ER, Satsangi J (2006). New genes in inflammatory bowel disease: lessons for complex diseases?. Lancet.

[125] Kaser A, Zeissig S, Blumberg RS (2010). Inflammatory bowel disease. Annu Rev Immunol.

